# A randomized controlled trial of gonadotropin-releasing hormone agonist versus gonadotropin-releasing hormone antagonist in Iranian infertile couples: oocyte gene expression

**DOI:** 10.1186/s40199-014-0067-4

**Published:** 2014-10-07

**Authors:** Fatemeh Sadat Hoseini, Seyed Mohammad Hossein Noori Mugahi, Firoozeh Akbari-Asbagh, Poopak Eftekhari-Yazdi, Behrouz Aflatoonian, Seyed Hamid Aghaee-Bakhtiari, Reza Aflatoonian, Nasser Salsabili

**Affiliations:** Department of Anatomy and Reproductive Biology, School of Medicine, Tehran University of Medical Sciences, Tehran, Iran; Departments of Histology, School of Medicine, Tehran University of Medical Sciences, Tehran, Iran; Department of Obstetrics and Gynecology, Tehran Women General Hospital, School of Medicine, Tehran University of Medical Sciences, Tehran, Iran; Department of Embryology at Reproductive Biomedicine Research Center, Royan Institute for Reproductive Biomedicine, ACECR, Tehran, Iran; Lab director Assisted Conception Units, Laleh Hospital, Tehran, Iran and Madar Hospital, Yazd, Iran; Department of Molecular Biology and Genetic Engineering, Stem Cell Technology Research Center, Tehran, Iran and Molecular Medicine Department, Biotechnology Research Center, Pasteur Institute of Iran, Tehran, Iran; Department of Endocrinology and Female Infertility at Reproductive Biomedicine Research Center, Royan Institute for Reproductive Biomedicine, ACECR, Tehran, Iran; Department of Physiotherapy, School of Rehabilitation of Tehran University of Medical Sciences, Tehran, Iran; Lab director Assisted Conception Unit, Tehran Women General Hospital, Tehran University of Medical Sciences, Tehran, Iran

**Keywords:** Gene expression, Controlled ovarian stimulation, GnRH antagonist, GnRH agonist

## Abstract

**Background:**

The main objective of the present work was to compare the effects of the gonadotropin-releasing hormone agonist (GnRH-a) and GnRH antagonist (GnRH-ant) on the gene expression profiles of oocytes obtained from Iranian infertile couples undergoing in vitro fertilization (IVF).

**Methods:**

Fifty infertile couples who underwent IVF between June 2012 and November 2013 at the Infertility Center of Tehran Women General Hospital, Tehran University of Medical Sciences, were included in this study. We included women that had undergone IVF treatment because of male factor, tubal factor, or unexplained infertility. The women randomly underwent controlled ovarian stimulation (COS) with either the GnRH-a (n = 26) or the GnRH-ant (n = 24). We obtained 50 germinal vesicle (GV) oocytes donated by women in each group. After the sampling, pool of 50 GV oocytes for each group was separately analyzed by quantitative polymerase chain reaction (qPCR).

**Result:**

The expression levels of Adenosine triphosphatase 6 (ATPase 6), Bone morphogenetic protein 15 (BMP15), and Neuronal apoptosis inhibitory protein (NAIP) genes were significantly upregulated in the GnRH-ant group compared to the GnRH-a group, with the fold change of 3.990 (SD ± 1.325), 6.274 (SD ± 1.542), and 2.156 (SD ± 1.443), respectively, (P < 0.001). Growth differentiation factor 9 (GDF9) mRNA did not have any expression in the GnRH-a group; however, GDF9 mRNA was expressed in the GnRH-ant group. Finally, it was found that the genes involved in the DNA repairing and cell cycle checkpoint did not have any expression in either group.

**Conclusion:**

The present study showed, for the first time, the expression levels of genes involved in the cytoplasmic maturity (BMP15, GDF9), adenosine triphosphate production (ATPase 6), and antiapoptotic process (NAIP), in human GV oocytes were significantly higher in the GnRH-anta group than in the GnRH-a group in COS. Higher expression level of these genes when GnRH-ant protocol is applied, this protocol seems to be a more appropriate choice for women with poly cystic ovarian syndrome, because it can probably improve the expression of the aforementioned genes.

**Trial registration:**

Current Controlled Trials: IRCT 2014031112307 N3.

## Background

Controlled ovarian stimulation (COS) is an important part of reproductive medicine; it also plays a vital role in inducing a pregnancy through assisted reproductive technology (ART). Higher pregnancy and implantation rates, compared to natural cycles, can be achieved using COS. Currently, three objectives are commonly followed when using COS for ART: ovulation induction, suppression of hypophyseal activity, and the growth stimulation of multiple follicles. For this purpose, two kinds of drugs are commonly used: gonadotropin releasing hormone agonists (GnRH-a) and gonadotropin releasing hormone antagonists (GnRH-ant). Multi-follicular recruitment causes a rapid increase in serum 17-beta estradiol (E2) levels during stimulated cycles, which in turn results in an untimely release of LH. The use of GnRH analogs can prevent the luteinizing hormone (LH) surge, which in turn improves the oocyte yield with more embryos and allows for better selection and therefore an increased pregnancy rate [1].

GnRH-a have been the most widely used drug for women undergoing COS, either for ICSI or for IVF [2]. GnRH-ant, on the other hand, has been introduced in clinical practice as a valid alternative in the last decade. In contrast to GnRH-a that decrease the number of receptors, GnRH-ant competitively inhibit endogenous GnRH from binding to its receptors. Consequently, they induce a direct, dose-dependent block of GnRH-receptors that are quickly reversible, which help avoid a flare effect [1].

Since 2001, several studies have compared the efficacy of the two GnRH analogs [2,3]. A recent Cochrane review indicated no evidence of a statistically significant difference in the rates of live births or ongoing pregnancies of the two GnRH analogs. In addition, the incidence of ovarian hyperstimulation syndrome (OHSS) in GnRH-ant treatment was lower than that of the GnRH-a treatment [3–5]. Furthermore, the following characteristics of the two protocols have also been compared: the number of oocytes retrieved and embryos transferred, the quality of oocyte morphology, implantation rate, the cycle cancellation rate, endometrial receptivity, follicular microenvironment, the percentage of granulosa cells with positive DNA fragmentation and apoptosis, genes expression in cumulus cells, and the distribution pattern and activity of human mature oocyte mitochondria [6–9].

It should be noted that the quality of oocytes obtained following controlled ovarian stimulation (COS) may vary significantly. Most oocytes are capable of being fertilized; however, nearly half of the fertilized ones can complete preimplantation development and even fewer ones can still implant. It has been shown that defects or variations in the ovulation or maturation processes have significant associations with gene expression alterations in oocytes and their supporting cells [10].

As mentioned earlier, although different clinical and molecular studies have been conducted to compare the efficacy of GnRH-a and GnRH-ant in assistant reproductive technique (ATR), results have been mostly inconsistent; Microarray studies conducted on human oocytes have indicated that some genes are expressed in both GV and MII stages, though with different levels. In fact, the more the oocytes move towards the maturity, the expression level of these genes increases [10–28]. These genes include those involved in the maturity of human oocytes (BMP15 and GDF9, both from the TGF beta category) [29,30], those involved in the cellular cycle and meiosis (BUB1, MAD2L1, CDC20, ATR, and ATM) [10–28,31], the energy-producing, mitochondrial gene (ATPase6) [27], and NAIP, which indicates oocytes viability [32–34]. Additionally, it is reported that COS can affect the gene expression level of oocytes [10]. During ovulation, mature and immature oocytes are obtained at the same time; however, the mature oocyte is used to treat patients. If differences are observed in the expression levels of the above mentioned genes in the cytoplasm of the GV oocytes due to different COS protocols (i.e. GnRH-ant or GnRH-a), the same conditions are expected to exist in the cytoplasm of mature oocytes. In other words, if increased levels of cytoplasmic maturity factors are observed following a COS protocol compared to the other, it highly likely to observe the same conditions in the cytoplasm of mature oocytes (MII) which co-exist with the GV oocytes in the same cycle. Therefore, for the first time, we decided to investigate the expression levels of nine genes involved in the cytoplasmic maturity, antiapoptotic process, cell cycle checkpoint, DNA repairing, and adenosine triphosphate production in germinal vesicle oocytes regarding the type of controlled ovarian stimulation in human. No studies have so far compared the genes expression in oocytes of women undergoing IVF/ICSI cycles between the GnRH-a and GnRH-ant protocols.

## Materials

### Subjects

Fifty infertile couples who underwent IVF/intracytoplasmic sperm injection (ICSI) between June 2012 and November 2013 at the Infertility Center of Tehran Women General Hospital, Tehran University of Medical Sciences, were included in this study. They were in good physical and mental conditions. We included the women that had undergone IVF treatment because of male factor, tubal factor, or unexplained infertility. These women did not have ovulatory dysfunction, were aged ≤40 years, and had a normal baseline follicle stimulating hormone (FSH) and luteinizing hormone (LH) (<10 mIU/mL).

## Methods

### Ovarian Stimulation and Oocyte Collection

The women underwent controlled ovarian stimulation with either the GnRH-a long protocol (n = 26) or the GnRH-ant fixed multi-dose protocol (n = 24), which was randomly assigned by the statistician [11,12].

The mean age (SD) of the participants was 31.7 (±5.7) years. In the GnRH-a long protocol group (n = 26), the treatment started by administering oral contraception pill (OCP) on the 2nd or the 3rd day of the pervious menstrual cycle. The daily administration of Buserelin acetate 500 μg (Suprefact, Aventis, Germany) was started preceding the IVF cycle from day 21 until pituitary down-regulation (serum E2 < 50 pg/ml in the absence of follicular structures larger than 10 mm). The Buserelin dose was reduced to 250 μg/d until the day of human chorionic gonadotropin (hCG) injection when pituitary down-regulation was achieved.

In the GnRH-ant fixed multi-dose protocol group (n = 24), Cetrorelix acetate 0.25 mg/day (Cetrotide, Serono, Switzerland) was initiated on the sixth day of the gonadotropin stimulation.

Ovarian stimulation was started on the 3rd day of the current menstrual cycle by injection of rFSH Follitropin alfa (Gonal F, Serono, Italy) at a daily dose of 150 to 225 IU in each group.

Administration of Buserelin and Cetrorelix was continued until hCG was injected. When at least 3 follicles with a mean diameter of 17 mm were developed (evaluated by transvaginal sonography), hCG 5000 IU/2/IM (Choriomon, IBSA Institut Biochimique S.A., Switzerland) was injected. About 34–36 h later, ultrasound-guided transvaginal oocyte retrieval was performed [13]. One hundred morphologically normal germinal vesicle oocytes were donated by 50 healthy women with normal ovarian reserve functions. The oocytes were aspirated transvaginally after COS. All the women had mature oocytes for ICSI, but they donated immature ones to our study. We obtained 50 germinal vesicle oocytes from 26 women aged 30.4 ± 5.5 years in the GnRH-a long protocol group and 50 germinal vesicle oocytes from 24 women aged 33.8 ± 5.6 years in the GnRH-ant protocol group. The oocytes were collected in a Quinn’s Advantage Medium with HEPES (Sage, USA) supplemented with 20 % human serum albumin and then granulosa cells were removed from oocytes using mechanical and chemical (Hyaluronidase type 4, Sigma Aldrich, USA) methods.

After stripping off granulosa cells, we used an inverted microscope (Nikon, Tokyo, Japan) to monitor the maturity of the oocytes.

In the COS cycles, immature oocytes constitute up to 10-15 % of the retrieved oocytes. Germinal vesicle (GV) oocytes are immature oocytes whose maturation process have been stopped in the prophase of the first meiotic stage and are characterized by enlarged nucleus and absent of polar body. Germinal vesicle oocytes were individually transferred into RNase-free micro centrifuge tubes. Then, 30 μl of RLT buffer (Ambion, Austin, USA) was added to them. All samples were kept in a refrigerator at a temperature of −80°C until the time of the analysis. After the sampling, the pools of 50 germinal vesicle oocytes from the GnRH-a long protocol group and 50 germinal vesicle oocytes from the GnRH-ant protocol group were separately analyzed by qPCR.

### RNA isolation, cDNA production and qPCR

ALLELEID 6.0 software was used for designing Exon-Junction primers. Molecular evolutionary genetics analysis (MEGA 4) software was also used for conducting sequence alignment. Oligo 6 Software was employed for the final assessment (Temperature/ Formation/False priming sites). Finally, we assessed primers in NCBI BLAST, as presented in Table 1.

**Table 1:** **Oligonucleotide primer sequences used for qPCR in the present study**

**Gene name**	**Primer**	**Accession no.**	**T°C**	**Product size (bp)**
***GDF9***		NM_005260.4	60	162
Sense	CCAGGTAACAGGAATCCTTC			
Antisense	GGCTCCTTTATCATTAGATTG			
***BMP15***		NM_005448.2	60	129
Sense	CCTCACAGAGGTATCTGGC			
Antisense	GGAGAGATTGAAGCGAGTTAG			
***ATPase 6***		YP_003024031.1	60	123
Sense	CTGTTCGCTTCATTCATTG			
Antisense	GGTGGTGATTAGTCGGTTG			
***NAIP***		NM_004536.2	60	184
Sense	GGAGTATTTGGATGACAGAAAC			
Antisense	TAGATTACCACTGGAGTCTTCC			
***BUB1***		NM_001278616.1	59	100
Sense	AAGGTCCGAGGTTAATCC			
Antisense	CACTGGTGTCTGCTGATAGG			
***MADL2***		NM_002358.3	60	169
Sense	CTTCTCATTCGGCATCAAC			
Antisense	ACACTTGTATAACCAATCTTTCAG			
***CDC20***		NM_001255.2	60	202
Sense	GATGTAGAGGAAGCCAAGATC			
Antisense	CCACAAGGTTCAGGTAATAGTC			
***ATR***		NM_001184.3	60	150
Sense	GATGCCACTGCTTGTTATG			
Antisense	CCACTCGGACCTGTTAGC			
***ATM***		NM_000051.3	60	107
Sense	GCATTACGGGTGTTGAAG			
Antisense	ATATAGAAGGACCTCTACAATG			
***β.actin***		NM_001101.3	60	90
Sense	CAAGATCATTGCTCCTCCTG			
Antisense	ATCCACATCTGCTGGAAGG			

Before isolating the RNA, the germinal vesicle oocytes were thawed within RLT buffer at room temperature and then pooled. To separate the RTL buffer from the pooled oocytes, they were then centrifuged at 12000 g for 3 minutes in order to extract total RNA, based on the standard protocol suggested by the manufacturer (Trizol, Invitrogen, USA). In order to remove genomic DNA contamination from the samples, the total RNA obtained from both groups was treated with DNase I (Fermentas, Sanktleon-rot, Germany). The total RNA concentration of the pooled germinal vesicle oocytes after treatment was 594 μg/ml for the GnRH-a long protocol group and 672 μg/ml for the GnRH-ant protocol group, determined by a Thermo Scientific Nano Drop 2000 Spectrophotometer. cDNA was synthesized according to manufacturer's instructions (Fermentas, Sanktleon-rot, Germany) using random hexamer primers.

We performed qPCR on the cDNA obtained from the pooled of germinal vesicle oocytes. Relative gene expression was calculated as the abundance ratio of each target gene to β-actin.

Quantitative real time PCR reactions were conducted in duplicates using a Roto- Gene Q instrument (Qiagen, German) with SYBR® Premix Ex Taq™ II master mix according to the procedure suggested by the manufacturer's instructions (Takara, Japan). The protocol for qPCR was initiated with a denaturizing step at 95°C for 30 seconds, followed by 50 cycles of 2-step, real-time PCR under the following conditions: 5 seconds at 95°C for denaturation and 30 seconds at 59–60°C for annealing and extension.

No template control (NTC) was used as the negative control. The specificity of the PCR fragments was determined using melting curve analysis. All melting curves produced one peak for each of the PCR products.

### Ethical considerations

The present study was approved by the ethics committee of Tehran University of Medical Sciences. The study was completely explained to the women, and informed consent was obtained before collecting germinal vesicles oocytes. The study was formally registered with the following code: IRCT 2014031112307 N3.

### Statistical analysis

We used One-way ANOVA to compare quantitative variables between the two groups and chi-square for qualitative variables by SPSS version 16 (Chicago, IL, USA). The significance level was set at 0.05. The efficiency values given by the Linreg software and relative expression were calculated using the REST 2009 software (Qiagen, Hilden, Germany) [14], which is a standalone software tool used for estimating up and down regulation for gene expression studies. The ΔΔCT was obtained by finding the difference between the groups. The fold change was calculated as FC = 2-^ΔΔCT^. For this purpose, β.actin was used as the reference gene for expression normalization.

## Results

There were no significant differences in the age, hormonal profile, number of oocytes retrieved, infertility duration, and cause of infertility between the two groups (P > 0.05). However, the serum level of 17-beta estradiol on the day of hCG administration was higher in the GnRH-ant protocol group than in the GnRH-a long protocol group; however, this difference was not statistically significant, as presented in Tables 2 and 3.

**Table 2:** **Mean (standard deviation) age, duration of infertility, number of oocytes retrieved, serum LH, FSH, TSH, PRL, AMH, and serum 17**-**beta estradiol in the GnRH-a protocol vs. GnRH-ant protocol group**

**Variable**	**GnRH-a long protocol (** ***n*** **= 26)**	**GnRH-ant protocol (** ***n*** **= 24)**	**Total (** ***n*** **=50)**	***P*** **value**
**Age (yrs)**	30.4 ± 5.5	33.8 ± 5.6	31.7 ± 5.7	0.100
**Duration of infertility (yrs)**	6.8 ± 3.5	4.6 ± 5.1	5.8 ± 4.5	0.086
**Retrieved oocytes no.**	11.0 ± 5.8	12.8 ± 8.3	11.8 ± 7.1	0.372
MII	6.2 ± 6.2	8.3 ± 6.3	7.2 ± 7.2	0.157
MI	1.8 ± 1.7	1.5 ± 1.6	1.6 ± 1.7	0.579
GV	2.1 ± 1.8	2.3 ± 1.8	2.2 ± 1.8	0.671
Deg.	0.8 ± 2.1	0.6 ± 2.1	0.7 ± 2.0	0.762
**Serum 17 beta-estradiol (Pg/ml)** *	5007.9 ± 8105	5988.3 ± 8110.6	5478.5 ± 8040.8	0.671
**Serum LH (IU/L)**	6.5 ± 2.9	5.5 ± 2.5	6.0 ± 2.8	0.256
**Serum FSH (IU/L)**	6.5 ± 2.5	6.3 ± 2.2	6.4 ± 2	0.706
**Serum TSH (μIU/L)**	3.0 ± 2.1	2.4 ± 1.1	2.7 ± 1.7	0.213
**Serum PRL (ng/ml)**	113.3 ± 186.2	107.0 ± 173.0	110.0 ± 178.2	0.902
**Serum AMH (ng/ml)**	5.0 ± 4.8	5.9 ± 5.6	5.4 ± 5.2	0.535

*On the day of hCG administration.

LH: luteinising hormone; FSH: follicle-stimulating hormone; TSH: thyroid-stimulating hormone; PRL: prolactin; AMH: anti-mullerian hormone.

MII: mature oocyte (Meiosis II); MI: immature oocyte (Meiosis I); GV: immature oocyte (Germinal Vesicle); Deg.: degenerative oocyte.

**Table 3:** **Distribution of the causes of infertility in the GnRH-a protocol vs. GnRH-ant protocol group**

**Variable**	**GnRH-a (%)**	**GnRH-ant (%)**	***P*** **value**
**Cause of infertility**			
● Male factor	16 (61.5)	9 (37.5)	
● Tubal factor	8 (30.8)	11 (45.58)	
● Unexplained	2 (7.7)	4 (16.7)	
**Total**	26.0 (100.0)	24.0 (100.0)	0.220

**Figure 1:**
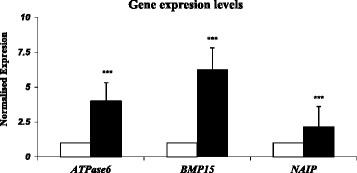
**Results of the gene expression analysis with REST when using β.actin as the reference gene.** Fold change (*Y* axis) represents the relative expression of *ATPase 6, BMP15, NAIP* mRNA in the pooled GV oocytes of the GnRH-ant protocol group (as tested group) versus the pooled GV oocytes of the GnRH-a long protocol group (as control group). ATPase 6, BMP15, and NAIP significantly were upregulated in GnRH-ant group in compared to GnRH-a group with the fold change of 3.990 (SD ± 1.325), 6.274 (SD ± 1.542), and 2.156 (SD ± 1.443), respectively, *** P < 0.001. Agonist protocol group □. Antagonist protocol group ■.

The present study showed that the expression levels of genes involved in the cytoplasmic maturity (*BMP15* and *GDF9)*, antiapoptotic process *(NAIP)*, and adenosine triphosphate production *(ATPase 6)* in human GV oocytes were significantly higher in the GnRH-ant group versus in the GnRH-a group. ATPase 6, BMP15, and NAIP were significantly upregulated in the GnRH-ant group compared to the GnRH-a group with the fold change of 3.990 (SD ± 1.325), 6.274 (SD ± 1.542), and 2.156 (SD ± 1.443), respectively, (P value < 0.001). *GDF9* mRNA did not have any expression in the GnRH-a group; however, *GDF9* mRNA was expressed in the GnRH-ant group. These results are shown in Figure 1.

Finally, it was found that the genes involved in the DNA repairing, i.e. Ataxia telangiectasia and Rad3-related protein *(ATR),* and Ataxia telangiectasia mutated *(ATM);* and those involved in the cell cycle checkpoint, i.e. Bone morphogenetic protein 15 (*BUB1),* Mitotic arrest deficient-like 1 (*MAD2L1)*, and Cell division cycle 20 (*CDC20),* did not have any expression in either group, as presented in Table 4.

**Table 4:** **The genes expression of germinal vesicle oocyte in GnRH agonist group compared with GnRH antagonist group**

**Gene symbol**	**Gene title**	**GnRH-a group**		**GnRH-anta group**	
		**Exist**	**Not exist**	**Exist**	**Not exist**
**Transforming growth factors**					
*GDF9*	Growth differentiation factor 9		+	+	
*BMP15*	Bone morphogenetic protein 15	+		+	
**Mitochondria**					
*ATPase6*	Adenosine triphosphatase 6	+		+	
**Antiapoptotic**					
*NAIP*	Neuronal apoptosis inhibitory protein	+		+	
**Cell cycle checkpoint markers**					
*BUB1*	Budding uninhibited by benzimidazoles 1		+		+
*MAD2L1*	Mitotic arrest deficient-like 1		+		+
*CDC20*	Cell division cycle 20		+		+
**DNA repair markers**					
*ATR*	Ataxia telangiectasia and Rad3		+		+
*ATM*	Ataxia telangiectasia mutated		+		+
**Reference gene**					
*B.actin*	beta actin	+		+	

## Discussion

Clinical studies have suggested similar pregnancy and live birth rates for both GnRH-a and GnRH-ant protocols [1,3,15–21], which has been further supported by some molecular studies [6–9]. Additionally, no significant morphological difference has been observed in the oocytes of the two groups after COS [22].

However, several advantages have been observed for the GnRH-ant protocol, including the fact that under this protocol, the endometrial receptivity is more similar to the natural cycle receptivity (in terms of endometrial chemokines and growth factors) [23]. In addition, when comparing GnRH-ant protocol and GnRH-a long protocols, the time of the appearance of the endometrial triple layer is statistically significant for the pregnancy rate only for the former protocol [24]. In addition to its safety and effectiveness, GnRH-ant allows for the flexibility of treatment in a wider range of women populations, including poor responders, women undergoing first-line controlled ovarian stimulation, and women diagnosed with polycystic ovarian syndrome. Therefore, the GnRH-ant protocol can be considered as a suitable alternative to the long agonist protocol, due mainly to its shorter duration of treatment and the need for fewer injections. Consequently, this leads to a significantly lower amount of administered gonadotropins, which most probably leads to improved women compliance [25]. We observed, for the first time, that the expression levels of genes involved in the cytoplasmic maturity, antiapoptotic process, and ATP production in human GV oocytes were significantly higher in the GnRH-anta group than in the GnRH-a group in COS.

As with other studies, in the present work, the same ovulation-triggering drug was used in the COS cycle for both groups; the only difference was the type of GnRH used for the two groups, which makes the present work different from the study of Hass et al. [26], in which oocyte cells were used for genetic evaluations.

The present study showed that the expression levels of genes involved in the cytoplasmic maturity, antiapoptotic process, and adenosine triphosphate production were significantly higher in the pooled oocytes of the women in the GnRH antagonist group versus those of the women in the GnRH agonist group (P < 0.001). These results are shown in Figure 1.

*ATPase 6* gene plays a critical role in ATP production by mitochondria. Deficiencies in the production of mitochondrial ATP can be linked to impaired oocyte fertilization, incomplete development of the embryo at later stages, and several other cellular and chromosomal disorders including errors in chromosomal segregation, lethal cytoplasmic defects, non disjunction disorders resulting in aneuploidy, and development failure of the sperm derived mitotic apparatus [27]. Therefore, the higher expression level of *ATPase6* in the pooled oocytes of the women in the GnRH-ant protocol group vs. those of the women in the GnRH-a long protocol group suggests that under the antagonist protocol, the mitochondrial activity may be more appropriate. In other words, higher-quality mitochondrial respiration and oxidative phosphorylation cascade occur in the oocytes of GnRH-ant group. The higher expression level of this gene and, in turn, higher energy production cause cell division spindles to form under better conditions [27].

Transforming growth factors beta *(TGF-ß)* are important paracrine growth factors that are secreted by the ovarian stroma or follicles surrounding the ovary, converting primordial follicles to primary ones. During folliculogenesis stages, oocytes secretion of *TGF-ß,* such as *BMP15* and *GDF9* [28], can regulate female fertility in several mammals [5,29,30]. *GDF9* and *BMP15* are responsible for transformation. They also cause the reproduction of granulosa cells under the influence of FSH, which mainly secrete estradiol [35]. Estradiol is required for the maturation of oocytes and development of embryo in vivo. Additionally, follicular atresia and granulosa cell apoptosis are inhibited by *GDF9*. Moreover, the proliferation, apoptosis, metabolism, and expansion of the cumulus oocyte complex are organized by the secretion of *GDF9* and *BMP15* [29].

According to the results from the present study, *GDF9* and *BMP15* are expressed in the pooled GV oocytes of the women in the GnRH-ant protocol group. Our study also showed that the expression level of *GDF9* was higher than that of *BMP15* in the antagonist group, which is consistent with the results of previous studies [30]. *GDF9* gene was not expressed in the pooled GV oocytes of the women in the GnRH-a long protocol group. The higher expression of these genes in the pooled GV oocytes of the GnRH-ant protocol group could be due to the fact that in the GnRH-a long protocol, complete inhibition of gonadotropins occurs on the 2nd or the 3rd day of the current menstrual cycle. The study of Lainas et al. [36] indicated that the serum levels of E2, FSH, and LH hormones were significantly higher before administering OCP on the 2nd or the 3rd day of the pervious menstrual cycle when compared to the same day in the OCP plus GnRH-a long protocol, mainly due to the suppression of inner gonadotropin in this protocol (FSH: 5.8 vs. 3.6 IU/L; LH:5.6 vs. 1.2 IU/L; and E2: 30.5 vs. 12 pg/ml before and after using OCP/GnRH-a, respectively). This, however, does not occur in the GnRH-ant protocol. In the GnRH-ant protocol, the initial dose of GnRH-ant is administered on the 6th or the 7th day of the current menstrual cycle, which causes immediate inhibition of LH by influencing the GnRH receptors in the pituitary gland. Therefore, adequate follicle stimulation can be provided by a combination of exogenous FSH and endogenous LH secretion in early treatment [37]. In other words, administration of GnRH-ant protocol occurs in late follicular development (on the 5th or the 6th day of the gonadotropin stimulation when estradiol is increased).

*NAIP* is one of the members of the inhibitor of apoptosis protein (IAP) family. By regulating the caspase activity (inhibition of both caspase 3 and caspase 9), which is an important part of the apoptotic machinery, this gene can prevent apoptosis of granulosa cells during the ovarian folliculogenesis stages and can cause the follicles to develop from the primary stage to the graafian follicle [32]. In the oocyte, the expression of this gene can increase 2–4 times due to the effect of gonadotropin, which indirectly leads to oocyte survival [33,34]. After a thorough literature search, it was determined that the present study examined, for the first time, the expression level of *NAIP* in human oocytes. The higher level of the expression of this gene in the pooled oocytes in the GnRH-ant protocol versus the pooled oocytes in the GnRH-a long protocol most likely suggests that the oocyte survival is improved by applying the former protocol.

In addition, in the present study, *BUB1, MAD2L1, CDC20, ATR, and ATM* genes did not have any expression in either group, which is most likely because that the oocytes used in the present work were germinal vesicle oocytes whose growth is arrested in the diplotene stage of the first meiotic prophase, as presented in Table 4.

*ATR* and *ATM* are a type of serine/threonine protein kinase. DNA double-strand breaks recruit and activate this serine/threonine protein kinase. This leads to the phosphorylation of several key proteins that are responsible for the initiation of DNA damage checkpoint activation, which finally results in cell cycle arrest, DNA repair, or apoptosis. *ATM* and *ATR* prevent premature chromosome condensation (PCC) until the DNA replication is completed [31].

In order to prevent premature separation of sister chromatids, *MAD2L1* and *BUB1*, which in turn interact with *CDC20* at check point activation, inhibit *CDC20/APC* [10]. Previous studies indicated that during the meiosis, *BUB1*, *MAD2L1*, and *CDC20* had high expressions in the oocytes [28].

There are controversial findings regarding the difference in the serum levels of estradiol on the day of hCG administration between GnRH-ant and GnRH-a long protocols. Some studies have reported that the serum levels of estradiol on the day of hCG administration are significantly higher in the GnRH-a long protocol than in the GnRH-ant protocol [38]; other studies, however, have reported reverse findings [21,39–41]. In the present study, the serum levels of estradiol on the day of hCG administration were higher in the GnRH-ant protocol than the GnRH-a long protocol although the difference was not statistically significant, as presented in Tables 2. The serum level of estradiol is an indicator of the function of granulosa cells, suggesting that these cells have better performance in producing estradiol in the GnRH-ant protocol versus the GnRH-a long protocol.

Studies have indicated that the application of GnRH agonist protocol causes mid-cycle gonadotropine flares and ovarian hyperstimulation syndrome (OHSS), which is more observed in women with poly-cystic ovarian syndrome (PCOs). Therefore, the GnRH-ant protocol is a better choice than the GnRH-a protocol for ovarian stimulation in infertile women due to PCOs [1,4,25].

On the other hand, Li et al. [30] reported that the expression of GDF9 and BMP15 genes is necessary for the maturity of the oocyte cytoplasm; therefore, these two can be used as markers of oocyte quality in terms of its evolution. In addition, it was reported that in the oocytes of infertile women with PCOs, the expression level of GDF9 and BMP15 genes is lower than that of normal individuals, which can be the reason for the lowered quality of oocytes in these patients, which in turn leads to reduced fertility and the rate of success in IVF.

Therefore, since the results from the present study suggested higher expression level of these genes when GnRH-ant protocol is applied [Figure 1], this protocol seems to be a more appropriate choice for women with PCOs, because it can probably improve the expression of the aforementioned genes.

Although similar studies should be performed on the matured oocytes, but obtaining of donated mature oocytes (in vivo) is not ethically feasible for research purposes. On the other hand, the mature oocytes obtained from GV in vitro maturation is not appropriate for this purpose, mainly because studies have indicated that the in vitro culture conditions have adverse genetic and epigenetic impact on the growth of GV oocytes; therefore, this can prevent the researchers from observing the same findings. We suggest that future studies should be performed on donated mature oocytes which, together with the results of the present work, can help provide a broader molecular perspective in this field and make the most appropriate ovarian stimulation protocol.

## Conclusions

Results from the present study indicated, for the first time, that in the germinal vesicle oocytes of women with normal ovarian function, the expression levels of genes involved in the cytoplasmic maturity (GDF9 and BMP15), antiapoptotic process (NAIP), and ATP production (ATPase 6) were significantly higher in the GnRH-anta protocol group than in the GnRH-a long protocol group. GDF9 mRNA did not have any expression in the GnRH-a group; however, GDF9 mRNA was expressed in the GnRH-ant group. Therefore, since the results from the present study suggested higher expression level of these genes when GnRH-ant protocol is applied, this protocol seems to be a more appropriate choice for women with PCOs, because it can probably improve the expression of the aforementioned genes.
